# Effects of hyperbaric therapy on liver morphofunctional of rabbits (*Oryctolagus cuniculus*) after hind limb ischemia-reperfusion injury

**DOI:** 10.14202/vetworld.2017.1337-1342

**Published:** 2017-11-14

**Authors:** Bambang Sektiari Lukiswanto, Wiwik Misaco Yuniarti, Y. Yosis Motulo

**Affiliations:** 1 Department of Veterinary Clinic, Faculty of Veterinary Medicine, Universitas Airlangga, Jl. Mulyorejo, Kampus C, Surabaya 60115, Indonesia; 2 Department of Surgery, Division of Thoracic, Cardiac, and Vascular Surgery, Dr. Soetomo General Hospital, Surabaya 60115, Indonesia

**Keywords:** hyperbaric oxygen therapy, ischemia, liver, morphofunctional, reperfusion

## Abstract

**Aim:**

The objective of this research was to study and to prove the effectiveness of hyperbaric oxygen therapy (HBOT) starting time on liver morphofunctional changes after ischemia-reperfusion in the hind limb of rabbits.

**Materials and Methods:**

This research used a complete randomized design with 4 groups and 6 repetitions on each. After 6 h artery femoral is ligation, reperfusion was performed for 100 min (G1), HBOT for 90 min after 10 min reperfusion (G2), 10 min reperfusion (G3), and HBOT 90 min after 60 min reperfusion (G4). Then, all of the rabbits were sacrificed. The liver and blood were taken for histopathological changes examination as well as for measuring the level of aspartate aminotransferase (AST) and alanine aminotransferase (ALT). The statistical test using Kruskal–Wallis and Mann–Whitney showed that the score of degeneration, necrosis, and portal inflammation in groups without HBOT (G1 and G3) were not significantly different, as well as in group with HBOT (G2 and G4) (p>0.05). However, the scores of histopathological changes in G1 and G3 were significantly different from those in G2 and G4 (p<0.05). The levels of AST and ALT in the groups without hyperbaric therapy (G1 and G3) were not significantly different from those in the groups treated with hyperbaric therapy (G2 and G4) (p>0.05).

**Result::**

Hind limb ischemia injury reperfusion can trigger damage for liver morphology, but not lead to liver dysfunction. Reperfusion can trigger increased activity of neutrophils, while neutrophil infiltration in the organ will lead to dysfunction. HBOT can inhibit the activity of neutrophils and the dysfunction of organs caused by ischemic reperfusion.

**Conclusion:**

HBOT for 90 min, both 10 and 60 min after the reperfusion, can protect hepatocytes from damage.

## Introduction

Ischemic condition can occur in a wide variety of disease status. Nevertheless, an ischemic condition often occurs in a variety of surgical procedures such as in a surgical process of vascular or organ transplantation. In such circumstances, ischemic injury can occur when blood flow to a tissue is reduced or stopped in a long time, resulting changes in the metabolism of muscles, from aerobic state into anaerobic one. Consequently, adenosine triphosphate (ATP) depletion and calcium (Ca) extracellular transfer into muscle cells occur, leading to abnormalities in metabolism and thrombosis, as well as tissue inflammation [1].

Blood flow improvement (reperfusion) is the main effort to overcome tissue ischemia (ischemia-reperfusion [IR]). However, reperfusion has negative side effects because it can cause damage and impaired organ function when it is not given appropriately. Inappropriate reperfusion then can trigger activation of proinflammatory mediators as well as formation of reactive oxygen species (ROS), resulting injury to the tissue [2]. In severe ischemic conditions, excessive inflammatory response after reperfusion can lead to a systemic inflammatory response syndrome and multiple organ dysfunction syndrome (MODS) [3]. MODS generally starts from the lungs and then spreads to the liver, gastrointestinal tract, and kidneys. Bone marrow and liver dysfunctions are in the final stages of MODS. Meanwhile, the dysfunction of the nervous system can occur at the beginning or end of MODS [4].

Many researchers actually have already been conducted on the effects of various drugs in reducing the negative impacts of reperfusion. Those previous researchers focused on the impacts of reperfusion used hyperbaric oxygen therapy (HBOT), a method of therapy using 100% oxygen with a high atmospheric pressure in a special room. HBOT has been proved to be very helpful to reduce the negative impacts of reperfusion in various tissues by reducing the activity of mediators of inflammation and oxidative stress induced by the reperfusion process [5].

Unfortunately, there have not been a lot of researchers aimed to know the appropriate time for the administration of HBOT in patients with various organ function disorders after reperfusion, especially its impact on liver organ. In patients with stroke, HBOT is known to be effective if given exactly 3 h after reperfusion since if given more than 3 h, it will not give any effect [6]. This research aimed to investigate effects of time-based HBOT administration on liver morphofunctional state of rabbits (*Oryctolagus cuniculus*) as animal models after hind limb reperfusion.

## Materials and Methods

### Ethical approval

This research was conducted under the ethical use of laboratory animals and also was approved by the Ethical Committee of Faculty of Veterinary Medicine, Universitas Airlangga.

### Research sites

This research was conducted at the Faculty of Veterinary Medicine, Universitas Airlangga, and also at the Department of Underwater and Hyperbaric Medicine, Hospital Dr. Ramelan, Surabaya. Preparations for histopathological examination and blood tests were made in the Department of Pathology and Veterinary Hospital, Faculty of Veterinary Medicine, Universitas Airlangga, Surabaya.

### Experimental animals

Experimental animals used in this research were 24 male New Zealand rabbits aged 6 months old and weighed 2-2.5 kg. Most studies use male animal models because of their stable hormonal status during treatment, and these animals are not at risk of being affected by hormonal (estrogen/progesterone) fluctuation related to the reproductive cycle that may confound the results of the study. Reperfusion injury using the rabbit as animal model showed significantly less infarct in female hearts as compared to males [7].

### Research materials

Materials used in this research were mineral water and food for rabbits, 70% alcohol, 10% ketamine, 2% xylazine hydrochloride, atropine sulfate, sterile thump, 10% buffered formalin, materials for staining hematoxylin-eosin (HE), materials for aspartate aminotransferase (AST) and alanine aminotransferase (ALT) examinations, and materials for the euthanasia of rabbits at the end of the research.

### Research tools

Tools used in this research were rabbit hutches, minor surgery equipment, masks, gloves, 10 cc syringe, syringes 3 cc, silastic band, hyperbaric chamber, a place to store organs, light microscope, object, cover glass, as well as blood container tube without anticoagulants.

### Experimental animal preparation

Those rabbits were adapted for 1 week before the research. They were fed and given to drink *ad libitum*. Next, femoral artery ligation was conducted on all the experimental animals. They were then divided into four groups, namely, G1, G2, G3, and G4.

Afterward, before anesthesia was conducted for surgery, all those rabbits were fasted for 12 h. Next, anesthesia was performed by weighing each rabbit and then injecting them with 0.02 mg/kg of atropine sulfate intramuscularly. After 10 min, anesthesia was carried out by injecting a combination of ketamine-xylazine with doses of 35 mg/kg and 5 mg/kg intramuscularly [7].

Although 30-50% of rabbits produce endogenous atropinase enzymes, because using atropine sulfate 0.1-0.2 mg/kg bw/sc, im. in some studies often helpful to decrease salivary production, bronchial secretion and preventing the occurrence of vagal which can cause bradycardia [8].

After all of those rabbits were anesthetized, an incision was made in the groin area of their sinister. Next, dissection was performed to separate the femoral artery from the nerve and the vein. Ischemia was induced by ligating the femoral artery with silastic band for 6 h, then followed with reperfusion by opening or removing the ligation to restore normal blood circulation in accordance with the treatment group.

Group 1 (G1): Femoral artery ligation for 6 h, followed with reperfusion by releasing ligation for 100 min (IR 100 min).

Group 2 (G2): Ligation for 6 h, followed with reperfusion by releasing ligation for 100 min. HBOT was given 10 min after the reperfusion for 90 min (IR 100 min with HBOT 90 min).

Group 3 (G3): Ligation for 6 h, followed with reperfusion by releasing ligation for 150 min (IR 150 min).

Group 4 (G4): Ligation for 6 h, followed with reperfusion by releasing ligation for 150 min. Hyperbaric therapy was given 60 min after the reperfusion for 90 min (IR 150 min with HBOT 90 min).

At the end of the research, all the rabbits were sacrificed. Their liver and blood were taken for examining histopathological changes in their liver using HE staining as well as for measuring the levels of AST and ALT. Histopathological changes were evaluated to assess the degree of liver damage as a result of the treatment given. Scoring method used in this research was a modification of Knodell method [9]. The type of the lesions observed and scoring technique used are as following:

**Table:** 

Type of lesions	Score	Note
Necrosis	0	No necrosis
	1	Necrosis on 1/3 of lobular
	3	Necrosis on 1/3-2/3 of lobular
	4	Necrosis on >2/3 of lobular
	5	Necrosis on 1/3-2/3 of lobular+bridging necrosis
	6	Necrosis on >2/3 of lobular+bridgingnecrosis
	10	Multilobular necrosis
Degeneration	0	No Degeneration
	1	Degeneration on 1/3 of lobular
	3	Degeneration on 1/3-2/3 of lobular
	4	Degeneration on >2/3 of lobular
Portal inflammation	0	No inflammation
	1	Light inflammation on 1/3 of lobular
	3	Mild inflammation on 1/3-2/3 of lobular
	4	Severe inflammation on >2/3 of lobular
Congestion	0	No hemorrhage
	1	Light (hemorrhage on 1/3 of lobular)
	2	Mild (hemorrhage on 1/3-2/3 of lobular)
	3	Severe (hemorrhage on >2/3 of lobular)

## Results

Scoring of histopathological changes in the livers was focused on necrotic lesions, degeneration, portal inflammation, and congestion. The results showed that within groups without hyperbaric therapy (G1 and G3), the scores of necrosis, degeneration, and portal inflammation were not significantly different (p>0.05). Similarly, within groups with hyperbaric therapy, namely, G2 and G4, the scores of necrosis, degeneration, and portal inflammation were not significantly different (p>0.05). However, the scores of histopathological changes in G1 and G3 were significantly different from those in G2 and G4 (p<0.05).

**Figure-1:**
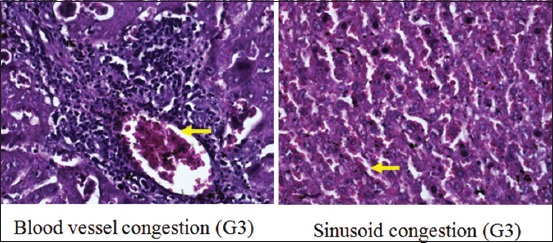
Congestion on group 3.

**Figure-2:**
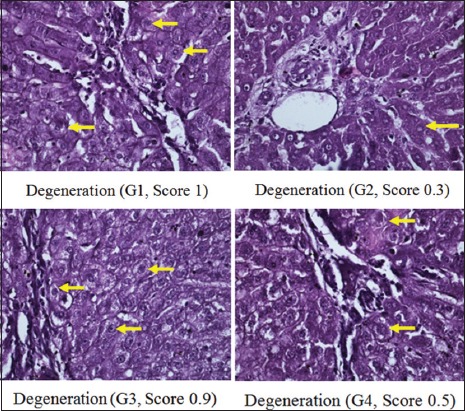
Degeneration of hepatocyte on each group.

**Figure-3:**
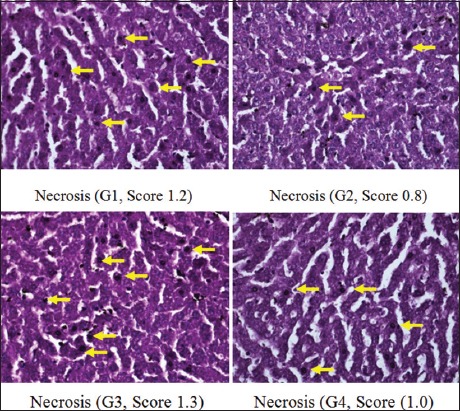
Necrosis of hepatocyte on each group.

**Figure-4:**
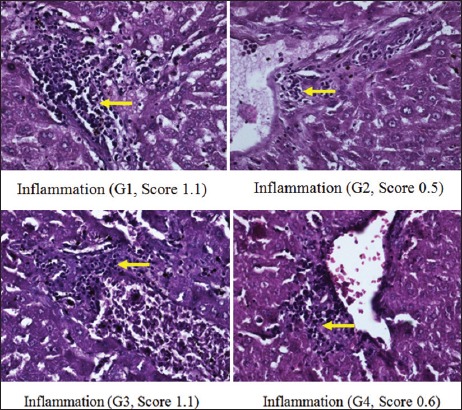
Portal inflammation on each group.

Moreover, scoring results of congestion degree had a different pattern, in which there was no congestion in G1, G2, and G4. Unlike the other three groups, congestion only appeared in G3 (p<0.05) (Figure-1). The degree of the total score (necrosis, degeneration, and portal inflammation) in the group without hyperbaric therapy was higher than in the group with hyperbaric therapy (Figures-2-4 and Table-1).

**Table-1:** Scoring results of histopathological changes in the livers (mean±SD) of each treatment group.

Treatment group	Necrosis	Degeneration	Inflammation portal	Congestion
G1	1.2^b^±0.2	1.0^b^±0.0	1.1^b^±0.6	0.0^a^±0.0
G2	0.8^a^±0.2	0.3^a^±0.1	0.5^a^±0.1	0.0^a^±0.0
G3	1.3^b^±0.17	0.9^b^±0.5	1.1^b^±0.6	0.3^b^±0.1
G4	1.0^a^±0.00	0.5^a^±0.1	0.6^a^±0.0	0.0^a^±0.0

Means with different superscripts letters are significant at p<0.05. SD=Standard deviation

Differences in the scoring results of histopathological changes between the groups without hyperbaric therapy, furthermore, did not seem affected by the length of reperfusion duration. For instance, there was no difference in histopathological scoring results and AST/ALT levels between Group G2 and Group G4 although both had different reperfusion duration, namely, 10 and 60 min before the administration of hyperbaric therapy.

In addition, results of the analysis of AST and ALT levels in each treatment group also showed a different pattern of results derived from the scoring results of histopathological changes in the livers. The results showed that the levels of AST and ALT in the groups without hyperbaric therapy (G1 and G3) were not significantly different from those in the groups treated with hyperbaric therapy (G2 and G4) (p>0.05) (Table-2). The levels of AST/ALT in all treatment groups, nevertheless, were still within normal values of AST/ALT levels in rabbits.

**Table-2:** Levels of AST and ALT in each group.

Treatment group	AST (IU/L)	ALT (IU/L)
G1	36.76^a^±3.11	12.60^a^±0.67
G2	38.08^a^±3.11	13.07^a^±0.67
G3	42.32^a^±3.11	12.05^a^±0.67
G4	40.07^a^±3.11	12.30^a^±0.67

Means with different superscripts letters are significant at p<0.05. AST=Aspartate aminotransferase, ALT=Alanine aminotransferase

## Discussion

Hepatic ischemia-reperfusion injury is a multifactorial pathophysiological process. Numerous studies have shown that this process includes excessive intracellular Ca, increased free oxygen radical, and increased of apoptosis or necrotic liver parenchymal cells [10,11]. The presence of oxidatives stress caused disturbances in the cell cycle and initiates apoptosis [12].

In this research, necrotic, degenerative, and inflammatory lesions on those livers were not accompanied with changes in the function of those livers by examining the levels of AST/ALT. This may be caused by the degree of hepatocyte damage classified into mild to moderate one. In addition, it can also be caused due to compensatory mechanism owned by those livers, where healthy hepatocytes would take over the function of hepatocytes suffering from the injury.

Similarly, a previous research also showed that limb ischemia suffered by mice for 2 h, then followed by reperfusion can cause injury to their lungs, but not in their liver and kidneys [13]. Unlike in the previous research, other researchers have also shown that limb ischemia suffered by mice for 3 h, then followed by reperfusion can cause injury to their lungs and kidneys, as well as the muscle of their lower limb [14,15].

Studies conducted directly with hepatic ischemia-reperfusion injury (HIRI) showed that this treatment cause hepatocytes apoptosis accompanied by elevate levels of AST and ALT, reflect severe hepatocyte damage [10,11]. These facts support an assumption that many negative effects of IR can trigger ischemia injury to vital organs near the ischemic area. Therefore, it can certainly be said that the improvement in the histopathological feature of G2 and G4 is not due to self-limited, but due to HBOT therapy given since IR was conducted 6 h after the induction of ischemia.

Vital organs, including liver, are very sensitive to ischemia that occurs on injury due to IR. Ischemia-reperfusion is usually accompanied by oxidative stress and then continued with stimulation of inflammatory response, eventually leading to injury to the liver [16]. Oxidation and inflammatory are mediators for the occurrence of injury in various organs after IR, which will end with a dysfunction of the organs, including liver [17].

Various factors, such as oxygen free radicals, migration and activation, abnormalities in the microcirculation, damage to endothelial cells sinusoid, coagulation cascade activation, and Kupffer cell activities to release inflammatory mediators and proteolytic enzymes, plays a role in causing injury to the liver due to IR [18,19]. Lesions on the liver caused by IR can occur in two phases, causing damage to hepatocytes. First, acute phase is characterized by the production of ROS by Kupffer cells, causing mild injury in the hepatocytes. In the next phase, there will be infiltration of leukocytes to the area of injury as a form of inflammatory cascade commencement. Neutrophil accumulation in the liver may cause injury to hepatocytes through oxidants and proteases released by neutrophils [20]. ROS after reperfusion in the initial phase is considered to have an important role in initiating and aggravating oxidative stress in the liver after reperfusion [21].

In this research, furthermore, HBOT could improve morphological function of liver, namely, in Group G2 and Group G4. On the other hand, according to Kihara [22], HBOT can trigger increased activity of neutrophils, while neutrophil infiltration in the organ will lead to dysfunction. In such conditions, HBOT can inhibit the activity of neutrophils, and the dysfunction of organs caused.

Da Silveira *et al*. [23] stated that the supply of oxygen after reperfusion can act as a protector of mitochondrial membrane. Oxygen is an agent that plays a role in the formation of ATP by oxidative phosphorylation. The energy generated is very necessary for the flow of both various ions out of the mitochondria as well as liquid into the mitochondria through the mitochondrial membrane. This process then will help to decrease the production and release of ROS, so the mitochondria can function normally. Therefore, an imbalance in this process can make oxidative damage become more extensive. This similar condition was also found in Group G1 and Group G3, experiencing ischemia in the same period without the administration of HBOT.

Biochemistry profile parameters, including AST/ALT, in rabbits, were influenced by various factors such as breed, age, sex, feed, liver disease, environmental conditions, stress, pregnancy, and heart rhythm [24-27]. Nevertheless, in this research, there was no difference in AST/ALT level between the rabbits without treatment and the rabbits with hyperbaric-treatment. In other words, all of those rabbits had no liver function impairment. Research on pigs was subjected left hemihepatectomy suggests that portal vein arterialization promotes early and improved liver regeneration without impairing liver function [28].

## Conclusion

Hind limb ischemia injury reperfusion can trigger a damage for liver morphology, but not lead to liver dysfunction. HBOT for 90 min, 10-60 min after the reperfusion, can protect hepatocytes from damage.

## Authors’ Contributions

BSL and YM carried out the main research works, WMY performed the statistical analysis and analyzed the main data in the experiments and BSL, WMY and YM approved the final manuscript. All authors read and approved the final manuscript.
